# Target Analysis and Retrospective Screening of Multiple Mycotoxins in Pet Food Using UHPLC-Q-Orbitrap HRMS

**DOI:** 10.3390/toxins11080434

**Published:** 2019-07-24

**Authors:** Luigi Castaldo, Giulia Graziani, Anna Gaspari, Luana Izzo, Josefa Tolosa, Yelko Rodríguez-Carrasco, Alberto Ritieni

**Affiliations:** 1 Department of Pharmacy, Faculty of Pharmacy, University of Naples “Federico II”, Via Domenico Montesano 49, 80131 Naples, Italy; 2 Department of Clinical Medicine and Surgery, University of Naples “Federico II”, Via S. Pansini 5, 80131 Naples, Italy; 3 Laboratory of Food Chemistry and Toxicology, Faculty of Pharmacy, University of Valencia, Av. Vicent Andrés Estellés s/n, Burjassot, València 46100, Spain

**Keywords:** mycotoxins, monitoring, pet food, HRMS-orbitrap, co-occurrence, retrospective screening

## Abstract

A comprehensive strategy combining a quantitative method for 28 mycotoxins and a post-target screening for other 245 fungal and bacterial metabolites in dry pet food samples were developed using an acetonitrile-based extraction and an ultrahigh-performance liquid chromatography coupled to high-resolution mass spectrometry (UHPLC-Q-Orbitrap HRMS) method. The proposed method showed satisfactory validation results according to Commission Decision 2002/657/EC. Average recoveries from 72 to 108% were obtained for all studied mycotoxins, and the intra-/inter-day precision were below 9 and 14%, respectively. Results showed mycotoxin contamination in 99% of pet food samples (*n* = 89) at concentrations of up to hundreds µg/kg, with emerging *Fusarium* mycotoxins being the most commonly detected mycotoxins. All positive samples showed co-occurrence of mycotoxins with the simultaneous presence of up to 16 analytes per sample. In the retrospective screening, up to 54 fungal metabolites were tentatively identified being cyclopiazonic acid, paspalitrem A, fusaric acid, and macrosporin, the most commonly detected analytes.

## 1. Introduction

Mycotoxins are a group of toxic secondary metabolites produced by fungi mainly belonging to *Aspergillus*, *Penicillium*, *Fusarium*, and *Alternaria* genera [1]. Due to the great structural diversity of these toxic compounds, they display a wide range of deleterious effects, including carcinogenic, hepatotoxic, nephrotoxic, teratogenic, heamatotoxic, immunotoxic, and hormonal or reproductive effects [2,3]. Mycotoxins pose a challenge to food safety as they are unavoidable and unpredictable contaminants in crops. In fact, the Food and Agriculture Organization (FAO) estimated that over one-quarter of the world’s food crop are contaminated with mycotoxins [4]. The mycotoxins with greatest agro-economic and health impact are aflatoxins (AFs), ochratoxin A (OTA), zearalenone (ZEN), fumonisins (FBs), and trichothecenes [5]. In the last decade, attention to the risk posed to human and animal health has also been extended to the so-called emerging *Fusarium* mycotoxins (including enniatins (ENNs) and beauvericin (BEA)) as well as the *Alternaria* toxins [6]. The factors affecting molds growth and/or mycotoxin production, and thus contamination of raw materials and feed, are associated with yield conditions (i.e., temperature, humidity, insect damage). Moreover, in post harvesting, other factors, such as moisture and storage conditions, could contribute to increasing risk of mycotoxin production [7].

Food crops susceptible to mycotoxin contamination include corn, wheat, barley, rye, rice, nuts, dried fruit, vegetables, and their derivatives [8]. It is remarkable that cereals and cereal by-products that are often unfit for human consumption are frequently used in feed formulations and act as excellent substrates for the fungal proliferation and production of mycotoxins. Recent surveys indicate that 70% of raw materials are contaminated with these toxins [9,10]. On the other hand, cereal processing, including dry milling, affects mycotoxin occurrence, especially for the fractions commonly designed for animal feeding [11,12]. Consequently, animal exposure to mycotoxins via plant-derived foods is of important consideration [13,14,15,16]. 

To limit the exposure to mycotoxins, the European Commission (EC) has set maximum limits of undesirable substances in both foodstuffs (EC/1881/2006 and amendments) and feedstuffs (2003/100/EC). As far as mycotoxins in feedstuffs are concerned, the Commission Directive 2003/100/EC has only established maximum admissible content of AFB1 in complete feedstuffs at 20 µg/kg. As regards the other mycotoxins, the European Union established in the Commission Decision 2006/576/EC guidance values regarding presence of deoxynivalenol (DON), ZEN, FBs, and OTA in products used as animal feeding (Table 1). 

In the last decades, improvement of analytical methods for the detection of mycotoxins at low ng/g range in a wide variety of foodstuffs has been performed [17]. Mass spectrometry-based techniques, such as MS and MS/MS, in combination with gas chromatography (GC) or liquid chromatography (LC) allowed the development of multi-mycotoxins methodologies [18]. Over recent years, there have been improvements in the LC-technique with the development of ultra-high-performance liquid chromatography (UHPLC), leading to higher peak efficiency and shorter chromatography run time [19]. In addition, the use of high-resolution mass spectrometry (HRMS), such as Orbitrap mass analyzers, is growing up in the ambit of food toxicology. HRMS analyzers have good specificity and high resolution due to mass accuracy provided by the resolution of Q-Orbitrap detectors combined with structural information obtained in MS/MS mode [20]. This technique enable the identification of untarget compounds and retrospective data analysis without the need to re-run samples. 

Even though investigations on mycotoxin distribution in feedstuffs are regularly conducted by competent authorities, the information on mycotoxin distribution of feedstuffs is limited [21]. Among the available studies focused on mycotoxins occurrence in feedstuffs, most of them have been performed in feed aimed to livestock production, whereas scarce literature have reported the occurrence of these toxic compounds in pet foods [22,23,24,25]. Therefore, the development and validation of analytical strategies to evaluate the occurrence of traditional and emerging mycotoxins in pet food to guarantee their quality, as well as to comply with trade requirements, are needed. Hence, the aim of this work was to develop an analytical tool based on a UHPLC-Q-Orbitrap HRMS method that combines quantitative target analysis for detection, quantification, and reliable identification of 28 mycotoxins from different fungi genera in pet food, with post-target screening (identification) of other 245 fungal and bacterial metabolites based on a comprehensive spectral library. In addition, the proposed methodology was applied to 89 dry commercially available pet food samples acquired from pet shops located in Campania region, Southern Italy.

## 2. Results and Discussion

### 2.1. Optimization of the Ultrahigh-Performance Liquid Chromatography Coupled to High-Resolution Mass-Spectrometry (UHPLC-Q-Orbitrap HRMS) Analysis

The optimization of the Q-Orbitrap HRMS parameters was performed via direct infusion of each mycotoxin standard (*n* = 28) diluted at 1 µg/mL into the Q-Orbitrap system using a flow rate of 8 µL/min. According to the literature, the addition of formic acid-ammonium formate shows better ionization efficiency of the studied analytes than acetic acid-ammonium acetate, and thus these additives were added to the mobile phases [26]. The most intense and signal stable adducts were selected for each analyte. Precursor ions were subjected to different values of collision energies (between 10 and 60 eV) to perform their fragmentation. Table 2 shows the UHPLC-HRMS parameters for the determination of mycotoxins included in this study.

On the other hand, three gradient programs were tested to achieve a good separation of the 28 mycotoxins: 

(i) Gradient 1: started with 20% B, kept up to 1 min, and then increased to 95% B in 1 min, followed by a hold-time of 0.5 min at 95% B. Afterward, the gradient switched back to 75% in 2.5 min, and decreased again reaching 60% B in 1 min. The gradient returned in 0.5 min at 20%, and 1.5 min column re-equilibration at 20%; 

(ii) Gradient 2: started with 10% B, kept up to 1 min, and then increased to 95% B in 1 min, followed by a hold-time of 0.5 min at 95% B. Afterward, the gradient switched back to 75% in 2.5 min, and decreased again reaching 60% B in 1 min. The gradient returned in 0.5 min at 10%, and 1.5 min column re-equilibration at 10%; 

(iii) Gradient 3: started with 0% B, kept up to 1 min, and then increased to 95% B in 1 min, followed by a hold-time of 0.5 min at 95% B. Afterward, the gradient switched back to 75% in 2.5 min, and decreased again reaching 60% B in 1 min. The gradient returned in 0.5 min at 0%, and 1.5 min column re-equilibration at 0%.

The results showed that several peaks eluted within the column dead time when starting the gradient program with high organic phase (20%, gradient 1) and the peak response was irregular. The second tested gradient (initial phase B set at 10%) decreased the number of analytes non-retained in the chromatographic column but still DON and its acetylated forms eluted within the first 1.0 min. The chromatographic separation of analytes was performed with a Luna Omega Polar C18 column. Optimal results in terms of retention time and good peak shape were achieved when the initial phase B was at 0%, obtaining good separation of the 28 mycotoxins in a total run time of 8 min (Table 2).

### 2.2. Optimization of Sample Preparation Procedure

Sample preparation has been recognized as a critical step in the chemical analysis workflow [27]. Few multi-mycotoxin methods have been reported in literature regarding pet food samples and most of them were performed with immunoaffinity column assays, increasing the cost of the method significantly [23,25,28]. Recently, a relatively cheap acetonitrile-based extraction was proposed in literature to determine seven *Fusarium* toxins in laboratory rat feed [11]. In this work, the sample preparation protocol reported by those authors was adopted as a starting point and slightly modified to extend it for the simultaneous determination of up to 28 target mycotoxins from different genera, including *Aspergillus, Penicillium, Fusarium,* and *Alternaria*. Critical extraction parameters were evaluated namely stirring time, sonication treatment, clean-up, and sample amount (Supplementary Table S1). All experiments were performed in triplicate using spiked samples at 20 µg/kg.

#### 2.2.1. Stirring Time

Three stirring times (15, 30, and 60 min) were tested to evaluate the effect of agitation in the extraction of mycotoxins. Results showed that 15 min of stirring time was not enough to reach acceptable recoveries (recovery range obtained for all mycotoxins: ≤40%) and RSD values (<23%). By increasing the stirring time up to 30 min, the recoveries for the wide majority of compounds increased (from 65 to 78%) except for AFs, for which recovery values lower than 55% were obtained. On the other hand, optimal results (recovery range: 72–105%, RSD < 16%) were achieved with 60 min of stirring for all studied compounds fulfilling the requirements set at Commission Decision EC 2002/657.

#### 2.2.2. Sonication Treatment

A sonication time of 15 min (with manual shaking every 5 min) was assayed and compared with samples in which the sonication step was not conducted. Results showed that when the sonication step was not performed, the accuracy and precision of the studied mycotoxins (recoveries ranging from 58 to 89%, RSD < 21%) were not as good as those obtained with sonicated samples (recoveries ranging from 72 to 114%, RSD < 14%); and therefore sonication treatment was included in the sample preparation procedure.

#### 2.2.3. Clean-Up Step

In the original method, a freeze-out step was carried out (minimum 2 h) to promote the precipitation of compounds that may interfere in the analysis based on the complexity of the samples [11]. Nonetheless, it significantly increases the time of the analysis. To overcome that, a clean-up step to reduce both matrix interferences and contamination of the instrument was evaluated. The efficiency of this strategy was evaluated by comparing the accuracy and precision data of the results obtained with samples stored in a freezer (2 h) and those submitted with a clean-up. According to literature, the mixture of 300 mg MgSO_4_ and 100 mg C18 (ratio 3:1, *w/w*) was selected as appropriate dispersive clean-up [16]. The results showed an improvement in accuracy and precision data due to the efficacy of the clean-up in removing interferences. Furthermore, the matrix effect was significantly minimized (range from 71 to 86%) with the addition of the clean-up, leading to an improved selectivity and robustness. In the samples in which the freezing out was conducted, impurities appeared. The usage of the clean-up step instead of freezing out made the extraction procedure faster and the extract obtained was much cleaner, as evidenced by the chromatographic response. 

#### 2.2.4. Sample Amount

Despite the significant reduction of interferences observed by the addition of a clean-up step, moderate signal suppression was obtained for most of the analyzed compounds, as specified in Section 2.2.3. To overcome that, the effect of reducing the sample amount was evaluated. Results showed that no matrix effect or slight signal suppression (≥85%) was obtained for all studied compounds when using 2 g of sample instead of 5 g, and therefore it allowed the quantification of the studied mycotoxins in pet food samples based on external calibration curves.

### 2.3. Method Validation

Calibration curves were prepared in triplicate at 8 concentration levels. Correlation coefficients (r^2^) greater than 0.9990 were obtained for all studied analytes within the linear range from limits of quantification (LOQs) to 1000 µg/kg. No matrix effect or slight signal suppression was observed for all mycotoxins ranging from 75 to 98%. Limits of detection (LODs) obtained were between 0.06 and 0.62 µg/kg; LOQs were calculated from 0.013 and 1.25 µg/kg, being lower than those reported in recent literature (Table 3). Average recoveries were in the range 75–112% for all studied mycotoxins at the fortification levels assayed (10, 20, and 100 µg/kg). Those results highlighted that the proposed methodology is accurate enough for the quantitative determination of the target mycotoxins. Intra-day and inter-day relative standard deviations (RSDs) showed reliable repeatability (RSD < 12%) and within-laboratory repeatability (RSD < 17%) of the developed method (Supplementary Table S2). The carry-over was evaluated by injecting a blank sample after the highest calibration point. No carry-over was present since no peaks were detected in retention time zone of all studied mycotoxins. In the Quality Assurance/Quality Control (QA/QC) procedure, the spiked sample was used in each sample batch in order to assess the accuracy and precision of the proposed method. To guarantee the quality of the results, every one of QA/QC criteria had to be achieved. To provide method reliability, satisfactory recoveries (between 70% and 120%, RSD < 20%) for all samples were required. When the results did not fit the expected criteria, the extractions were repeated in order to achieve this range. After the optimization and validation procedure and during the sample analysis, none of the QA/QC samples were outside of the expected criteria in any batch of samples.

### 2.4. Occurrence of Mycotoxins in Pet Food Samples

The optimized and validated multi-mycotoxin method was applied to 89 dry pet food samples (55 for dogs and 34 for cats) acquired from different pet shops located in Campania region, Southern Italy. Table 3 shows the results here obtained, as well as reviewing the available studies published in the last decade regarding the occurrence of mycotoxins in pet food samples.

In these analyzed samples, 99% of pet foods showed mycotoxin contamination. Despite the significantly high incidence, the concentration levels found were below the maximum level and/or maximum permissible levels set for mycotoxins in feedstuffs (2003/100/EC; 2006/576/EC). Nonetheless, special attention must be considered for the aflatoxins group. 25.8% of analyzed samples showed AFB1 contamination in a concentration range from 3.3 to 7.9 µg/kg (average content: 4.3 µg/kg). Similar AFB1 findings were reported in petfoods from Poland (*n =* 49) in a concentration range from <LOQ to 0.2 µg/kg [22], and from Brazil (*n* = 180) with AFB1 contamination levels ranging from 0.3 to 9.4 µg/kg [31]. In another survey, 4 out of 70 Brazilian pet food samples showed AFB1 contamination in a range from 15 to 37 µg/kg. This high contamination level reported by these authors was related to the presence of contaminated peanuts present in all positive samples. Despite some samples exceeded the maximum limit set by the EU for complete feedstuffs (20 µg/kg), those levels were below the permitted limits adopted in Brazil (50 µg/kg).

On the other hand, OTA was quantified in 2.2% of the here analyzed pet food samples at average content of 1.5 µg/kg. These levels are in agreement with previous studies as reviewed in Table 3. However, a wide range of OTA incidence reported by the different surveys was observed. Concerning the occurrence of fumonisins in pet food samples, the available studies reported both high incidence (>50%) and concentrations up to hundreds/thousands µg/kg (Table 3). Recently, Teixeira et al. [25] reported FBs contamination in 70% (*n* = 87) of Brazilian pet food samples at a concentration range from 30 to 1015 µg/kg. These findings are also in line with the data here obtained in which 67% of analyzed samples showed FBs contamination ranging from 10.5 to 990.1 µg/kg, being FB1 the most commonly detected fumonisin. The particularly high levels and incidence of FBs in feed could be related to the quality of corn (grain) and corn-based ingredients used in the formulations of these feedstuffs.

As far as trichothecenes are concerned, DON (and its acetylated forms) were the most commonly reported type B trichothecene in pet food samples reported in literature at concentration levels of hundreds µg/kg (Table 3). Similar results were here found; in fact, 30% of samples were DON-contaminated at concentration range from 7.6 to 297.3 µg/kg. On the other hand, type A trichothecenes mainly represented by HT-2 and T-2 toxins, has been barely investigated in pet food samples despite the fact that these toxins have been proven to have a higher toxicity than DON. In these analyzed samples, HT-2 (32.6% positive samples) and T-2 (47.2% positive samples) were detected at levels from 3.3 to 110.1 µg/kg, and from 0.7 to 9.0 µg/kg, respectively. Higher HT-2 and T-2 incidences (of up to 87.7%) than those here obtained were reported by Błajet-Kosicka et al. [22], in the 49 Polish pet food samples, but the concentration levels in that study were below 20 µg/kg in all positive samples. In line with that, ZEN (and its derivative forms) were found in 91% of the here analyzed samples at levels ranging from < LOQ to 60.6 µg/kg. These results are in agreement with recent surveys carried out in Brazilian [23,25], Egyptian [21], Polish [22], and Austrian [30] pet food samples (Table 3).

Emerging *Fusarium* mycotoxins (ENs and BEA) and *Alternaria* mycotoxins (AOH and AME), have been barely investigated in feed samples. The results showed a high incidence (>80%) of enniatins with concentration up to hundreds µg/kg (Table 3). Among enniatins, ENNB was the most commonly detected mycotoxin in the assayed samples (83 out of 89). The results obtained in this work are according to contents reported in different feedstuffs samples. Tolosa et al. [32] reported a high incidence of ENs (100% positive samples) and BEA (95% positive samples) in 20 Spanish fish feed at levels ranging from 0.1 to 10.0 µg/kg and from 0.1 to 6.6 µg/kg respectively. These results are also according to those reported by Warth et al. [33] in which ENs and BEA were present in 70% and 100% (*n* = 10) of animal feed samples from Burkina Faso and Mozambique, with concentration levels ranging from 0.1 to 114.0 µg/kg and from 3.3 to 418 µg/kg, respectively. On the other hand, Warth et al. [33] reported also AOH and AME contamination in 75% and 25% in a low number of samples analyzed (*n* = 4) at average content of 15.1 and 11.1 µg/kg, respectively. Similar high incidence was reported by Streit et al. [34], in which AOH and AME were found in 80% and 82% of feed and feed raw materials (*n* = 83) from Europe at concentration levels of hundreds µg/kg.

### 2.5. Co-occurrence of Mycotoxins in Analyzed Samples

**Figure 1:**
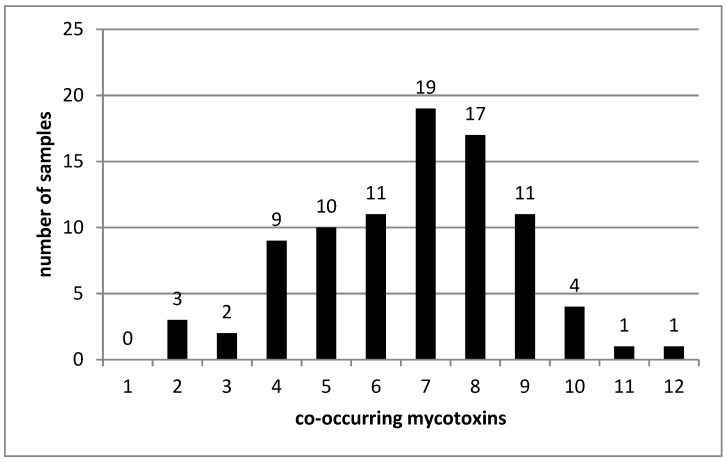
Number of samples co-contaminated with a given number of mycotoxins (total samples analyzed; *n =* 89).

All contaminated pet food samples here analyzed showed co-occurrence from three to sixteen mycotoxins in a sum concentration range from 1.6 to 1700.0 μg/kg (Figure 1). Three multicontaminated samples showed sum concentrations above 1000 μg/kg, with several *Fusarium, Aspergillus* and *Alternaria* toxins. A significant number of pet food samples (77.3%) were co-contaminated from 8 to 12 mycotoxins. Similarly, Böhm et al. [30] reported the co-occurrence of mycotoxins in 33% Austrian pet food samples (*n* = 76), and *Fusarium* toxins such as DON, ZEA, and FBs were the most predominant. The simultaneous occurrence complicates the evaluation of toxicological potential of feed. Additive and synergistic effects on overall toxicity are frequently observed when mycotoxin mixtures are evaluated [9,35,36].

### 2.6. Identification of Non Target Coumpounds Based on A Retrospective Screening Analysis

**Figure 2:**
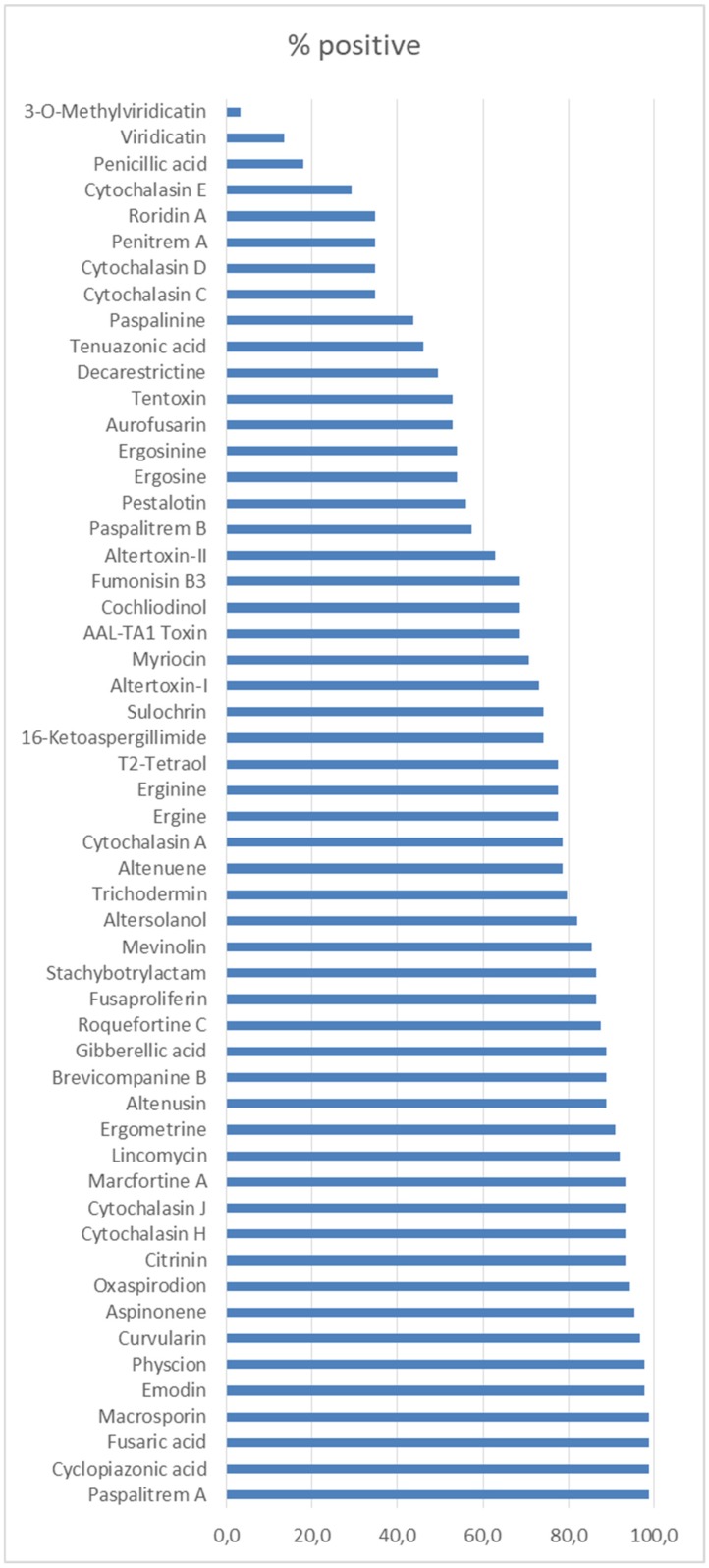
Non-target mycotoxins identified in samples based on ultrahigh-performance liquid chromatography coupled to high-resolution mass-spectrometry (UHPLC-Q-Orbitrap HRMS) library.

The developed strategy based on Q-Orbitrap HRMS combines the quantitative target determination with the post-target screening approach. The possibilities of the Q-Orbitrap HRMS were further explored by subjecting the full scan data of the pet food samples to untargeted screening with the major data processing parameters set as follows: ionization patterns [M + H]^+^ and [M − H]^−^, a minimum peak area of 1 × 10^5^ a.u., a maximum mass window of 5 ppm, and a retention time width of 1 min. The confirmation of the structural characterization of unknown compounds and untargeted analytes was based on the accurate mass measurement, elemental composition assignment, and MS/MS spectrum interpretation. Untargeted data processing was carried out using structural formula finder tool and the online high-quality mass spectral database. The advantage of using a full-scan acquisition mode is to allow the retrospective analysis of samples for the identification of up to 245 fungal and bacterial metabolites included in spectral library database by processing the raw data of the analyzed pet food samples. Fifty-four fungal metabolites were tentatively identified in the here analyzed samples (Figure 2). Cyclopiazonic acid, paspalitrem A, fusaric acid, and macrosporin were the most commonly detected mycotoxins in the assayed samples (98.9%). Cyclopiazonic acid and paspalitrem A are produced by *Aspergillus* and *Penicillium* spp. Fusaric acid is produced by some *Fusarium* spp. Macrosporin is mainly produced by *Stemphylium* spp. These metabolites have been already found in contaminated cereal crops such as oats, barley, millet, corn, and rice [37,38,39]. In these analyzed samples, emodin was also identified in 97.8% of feed samples. This compound was already reported in Spanish feed and feed raw material but lower incidence (57.1%; *n* = 62) [40].

## 3. Conclusions

A UHPLC-Q-Orbitrap HRMS method for simultaneous determination of mycotoxins from different fungal species in pet food samples was in-house optimized and validated according to the criteria set by Commission Decision 2002/657/EC. In addition, mycotoxin spectral library of 245 analytes was used for post-run retrospective screening. The developed method was successfully applied to eighty-nine petfood samples and twenty-six different mycotoxins were found at high incidence (98.9%) but at concentrations below the maximum permissible limits. Co-occurrence of mycotoxins was found in all contaminated samples with up to sixteen analytes per sample. The established method was rapid and efficient, and capable of covering more analytes compared to the previous methods for the detection and quantitation of mycotoxins in pet food products. Moreover, this is the first work describing the simultaneous detection, quantification, and retrospective screening of a wide range of mycotoxins from different genera in pet food samples by using the capability provided by the UHPLC-Q-Orbitrap HRMS technology.

## 4. Materials and Methods 

### 4.1. Chemical and Reagents

Mycotoxin standards and metabolites namely aflatoxins (AFB1, AFB2, AFG1, and AFG2), ochratoxin A (OTA), fumonisins (FB1 and FB2), deoxynivalenol (DON), 3-acetyl-deoxynivalenol (3-AcDON), 15-acetyl-deoxynivalenol (15-AcDON), HT-2 toxin, T-2 toxin, neosolaniol (NEO), diacetoxyscirpenol (DAS), fusarenon-X (FUS-X), zearalenone (ZEN), α-zearalenol (α-ZEL), β-zearalenol (β-ZEL), α-zearalanol (α-ZAL), β-zearalanol (β-ZAL), zearalanone (ZAN), beauvericin (BEA), enniatins (ENNA, ENNA1, ENNB, and ENNB1), alternariol (AOH), and alternariol monomethyl ether (AME) were purchased from Sigma Aldrich (Milan, Italy). Individual stock solutions of all analytes were prepared by diluting 1 mg of each mycotoxin in 1 mL of methanol and further diluted for preparing working standard solutions. All these solutions were kept in safe conditions at −20 °C.

All solvents, acetonitrile (AcN), methanol (MeOH) and water (LC-MS grade) were purchased from Merck (Darmstadt, Germany) whereas formic acid (mass spectrometry grade) and ammonium formate (analytical grade) were obtained from Fluka (Milan, Italy). Magnesium sulphate was obtained from VWR Chemicals BDH Prolabo, (Leuven, Belgium) and C18 (analytical grade) was purchased from Supelco (Bellafonte, Pennsylvania, PA, USA). 

Syringe filters with polytetrafluoroethylene membrane (PTFE, 15 mm, diameter 0.2 µm) were provided by Phenomenex (Castel Maggiore, Italy); conical centrifuge polypropylene tubes of 50 mL and 15 mL were obtained from BD Falcon (Milan, Italy).

### 4.2. Sampling

A total of eighty-nine standard dry pet food samples was randomly purchased from different pet shops located in Campania region, Southern Italy. The acquired pet food samples were classified as follows: dogs (*n=* 55) and cats (*n =* 34). The nutritional composition of the analyzed samples is shown in Table 4. The main ingredients declared in labels from samples were rice, corn, corn flour, wheat, tapioca, wheat flour, oat, and barley. All samples were homogenized using a laboratory mill (particle size 200 µm) and then stored in a dark and dry place until analysis. The analysis was performed within 3 days after sample registration [40].

### 4.3. Sample Preparation

In this work, a sample preparation procedure for extraction of mycotoxins from laboratory rat feed reported in literature was selected as starting point and slightly modified [11]. In brief, homogenous representative samples (2 g) were weighted into 50 mL falcon tube and 10 mL of AcN:H_2_O mixture (80:20, *v/v* with 0.1% of formic acid) were added. The mixture was placed in a horizontal shaker for 60 min at 245× *g* and then placed into an ultrasonic bath for 15 min. Samples were centrifuged for 3 min at 3435× *g* at 4 °C, and 2 mL of the upper layer were submitted to a dispersive-SPE with a mixture of 300 mg of anhydrous MgSO_4_ and 100 mg of C18, and vortexed for 1 min. The mixture was centrifuged for 1 min at 1472× *g* at 4 °C. Finally, the extract was evaporated to dryness under gentle nitrogen flow at 45 °C, reconstituted with 0.5 mL of MeOH/H_2_O (70:30, *v/v*), and filtered (0.22 µm filter) prior to the UHPLC-Q Orbitrap HRMS analysis.

### 4.4. Method Validation

The method validation was performed in-house with respect to linearity, matrix effect, sensitivity, accuracy, and precision, as expected with compliance to Commission Decision 2002/657/EC. For the spiking and recovery studies, there were employed a pool of blank pet food samples (*n =* 10) (dog, *n =* 5; and cat, *n =* 5) of previous studies. Linearity was evaluated throughout standard solutions and matrix-matched calibrations. A graphic scatter plot test was used to assess the linearity, and lack-of-fit test was performed in linear regression model. Linear range of the method was assessed from limit of quantification to 1000 µg/kg for all mycotoxins. Matrix effect was evaluated by comparing the slopes of standard solutions built in neat solvent and the matrix-matched calibration curve. Values around 100% mean that there are no matrix effects, signal suppression, or enhancement if the value obtained was lower or higher than 100%, respectively. The sensitivity was evaluated by LODs and LOQs. LOD was defined as the minimum concentration where the molecular ion can be identified with a mass error below 5 ppm, and LOQ was set as the lowest concentration of the analyte that produce a chromatographic peak with precision and accuracy <20%. The accuracy of the method was evaluated with recovery studies. Blank samples were spiked and left to equilibrate overnight and then extracted as previously described. Method recovery was performed at three spiking levels (10, 20, and 100 µg/kg). Precision was expressed as relative standard deviation (% RSD) and calculated by triplicate measurements carried out on a single day (repeatability) and on three non-consecutive days (within-laboratory repeatability) [41].

### 4.5. Quality Assurance/Quality Control 

For the confirmation criteria, the peaks for the studied compounds in the samples were confirmed by comparing the retention times of the peak with those of standard solutions at a tolerance of ± 2.5%. To ensure a higher level of confidence in the identification, the precursors and product ions were recognized with a mass error below 5 ppm. In the QA/QC procedure, a sample blank, a reagent blank, a replicate sample, and a matrix-matched external calibration were added at the beginning and end of each sample batch in order to assess the effectiveness of the developed method. Spiked pet food samples at three concentration levels (10, 20, and 100 µg/kg) were used for analytical quality control.

### 4.6. Ultrahigh-Performance Liquid Chromatography Coupled to High-Resolution Mass-Spectrometry (UHPLC-Q-Orbitrap HRMS) Analysis

Detection and quantitation were performed with a UHPLC instrument (Dionex Ultimate 3000, Thermo Fisher Scientific, Waltham, Ma, USA) equipped with a degassing system, a Quaternary UHPLC pump working at 1250 bar, and an autosampler device. Chromatographic separation of analytes was performed with a thermostated Luna Omega Polar C18 column (50 × 2.1 mm, 1.6 µm, Phenomenex) kept at 30 °C. Both mobile phases contained 0.1% formic acid and 5 mM ammonium formate and were H_2_O (phase A) and MeOH (phase B). The LC gradient started with 0% B, kept up to 1 min, and then increased to 95% B in 1 min, followed by a hold-time of 0.5 min at 95% B. Afterward, the gradient switched back to 75% in 2.5 min, and decreased again reaching 60% B in 1 min. The gradient returned in 0.5 min at 0%, and 1.5 min column re-equilibration at 0%. The injection volume was 5 µL with flow rate of 0.4 mL/min. The UHPLC system was coupled to a Q-Exactive Orbitrap mass spectrometer (UHPLC, Thermo Fischer Scientific, Waltham, Ma, USA). The mass spectrometer was operated in both positive and negative ion mode using fast polarity switching by setting two scan events (Full ion MS and All ion fragmentation, AIF). Full scan data were acquired at a resolving power of 35,000 FWHM at *m/z* 200. The conditions in positive ionization mode (ESI^+^) were: spray voltage 4 kV; capillary temperature 290 °C; S-lens RF level 50; sheath gas pressure (N_2_ > 95%) 35, auxiliary gas (N_2_ > 95%) 10, and auxiliary gas heater temperature 305 °C. Ion source parameters in negative (ESI^−^) mode were: spray voltage −4 kV; capillary temperature 290 °C; S-lens RF level 50; sheath gas pressure (N_2_ > 95%) 35, auxiliary gas (N_2_ > 95%) 10, and auxiliary gas heater temperature 305 °C. Value for automatic gain control (AGC) target was set at 1 × 10^6^, a scan range of *m/z* 100–1000 was selected and the injection time was set to 200 ms. Scan-rate was set at 2 scans/s. For the scan event of AIF, the parameters in the positive and negative ion mode were: mass resolving power = 17,500 FWHM; maximum injection time = 200 ms; scan time = 0.10 s; ACG target = 1 × 10^5^; scan range = 100–1000 *m/z*, isolation window to 5.0 *m/z*, and retention time window to 30 *s*. The collision energy was optimized individually for each compound. Different collision energies were tested while the infusion of the compound was performed into the HRMS. The optimal energy was chosen when at least the parent compound remained at 10% intensity and it produced characteristic product ions from 80–100% intensity. Data processing were performed by the Quan/Qual Browser Xcalibur software, v. 3.1.66. (Xcalibur, Thermo Fisher Scientific). Retrospective screening was carried out on spectral data collected using a mycotoxin spectral library (Mycotoxin Spectral Library v1.1 for LibraryView™ Software, AB SCIEX, Framingham, USA). The identification was based on accurate mass measurement with a mass error below 5 ppm for the molecular ion; while regarding the fragments on the intensity threshold of 1000 and a mass tolerance of 5 ppm. Quantitative results were obtained working in scan mode with HRMS exploiting the high selectivity achieved in full-scan mode, whereas MS/HRMS information was used for confirmatory purposes.

### 4.7. Statistics and Data Analysis

All validation experiments were performed in triplicate, and the results were expressed as the average values ± relative standard deviation (RSD, %). Student’s *t*-test statistical analysis was performed for data evaluation; *p* values < 0.05 were considered significant.

## Figures and Tables

**Table 1:** Regulated and recommended maximum levels of mycotoxins in feed materials set by the European Commission.

Mycotoxin	Products	^a^ Regulated Maximum Level (mg/kg) Relative to a Feedingstuff	^b^ Guidance Value (mg/kg) Relative to a Feedingstuff	^c^ Guidance Value in mg/kg (ppm) Relative to a Feedingstuff with a Moisture Content of 12 %
AFB1	All feed materials; complete feedingstuffs for pigs and poultry (except young animals); complementary feedingstuffs for cattle, sheep and goats (except complementary feedingstuffs for dairy animals, calves and lambs); complementary feedingstuffs for pigs and poultry (except young animals); complete feedingstuffs for cattle, sheep and goats with the exception of:	0.02		
	complete feedingstuffs for dairy animals	0.005		
	complete feedingstuffs for calves and lambs	0.01		
DON	Maize by-products		12	
	Other cereals and cereal products		8	
	Complementary and complete feedingstuffs with the exception of:		5	
	complementary and complete feedingstuffs for pigs		0.9	
	complementary and complete feedingstuffs for calves (<4 months), lambs and kids		2	
ZEN	Maize by-products		2	
	Other cereals and cereal products		3	
	Complementary and complete feedingstuffs for piglets and young sows		0.1	
	Complementary and complete feedingstuffs for sows and fattening pigs		0.25	
	Complementary and complete feedingstuffs for calves, dairy cattle, sheep and goats		0.50	
OTA	Cereals and cereal products		0.25	
	Complementary and complete feedingstuffs for pigs		0.05	
	Complementary and complete feedingstuffs for poultry		0.1	
FBs	Maize and maize products		60	
	Complementary and complete feedingstuffs for pigs, horses, rabbits and pet animals		5	
	Complementary and complete feedingstuffs for fish		10	
	Complementary and complete feedingstuffs for poultry, calves (<4 months), lambs and kids		20	
	Complementary and complete feedingstuffs for adult ruminants (>4 months) and mink		50	
T-2 + HT-2 toxin	Compound feed for cats			0.05

^a^ Directive Commission 2003/100/EC; ^b^ Recommendation Commission 2006/576/EC; ^c^ Commission Recommendation 2013/637/EU; Abbreviations: AFB1: aflatoxin B1; DON: deoxynivalenol; ZEN: zearalenone; OTA: ochratoxin A; FBs: fumonisins (FB1 and FB2).

**Table 2:** Ultrahigh-performance liquid chromatography coupled to high-resolution mass-spectrometry (UHPLC-HRMS) parameters for the determination of mycotoxins included in this study.

Mycotoxins	Retention Time (min)	Elemental Composition	Adduct Ion	Theoretical Mass (*m/z*)	Product Ion	Collision Energy (eV)
AFB1	4.64	C_17_H_12_O_6_	[M + H]^+^	313.07066	285.07489; 269.04373	36
AFB2	4.98	C_17_H_14_O_6_	[M + H]^+^	315.08631	287.09064; 259.05945	36
AFG1	4.79	C_17_H_12_O_7_	[M + H]^+^	329.06558	243.06467; 200.04640	40
AFG2	4.61	C_17_H_14_O_7_	[M + H]^+^	331.08123	313.07010; 245.08032	37
OTA	6.50	C_20_H_18_NO_6_Cl	[M + H]^+^	404.08954	358.08304; 341.05658	16
FB1	6.03	C_34_H_59_NO_15_	[M + H]^+^	722.39575	352.32010, 334.30963	48
FB2	6.78	C_34_H_59_NO_14_	[M + H]^+^	706.40083	336.32547; 318.31488	58
DON	4.18	C_15_H_20_O_6_	[M + HCOOH]^−^	341.12451	295.1189; 265.10822	−12
3-ADON	3.83	C_17_H_22_O_7_	[M + H]^+^	339.14383	231.10118; 203.10638	20
15-ADON	4.02	C_17_H_22_O_7_	[M + H]^+^	339.14383	261.11154; 137.05957	20
HT-2	5.63	C_22_H_32_O_8_	[M + NH_4_]^+^	442.24354	263.12744; 215.10641	27
T-2	6.13	C_24_H_34_O_9_	[M + NH_4_]^+^	484.25411	215.10603; 185.09561	23
NEO	4.32	C_19_H_26_O_8_	[M + NH_4_]^+^	400.19659	305.13803; 141.0053	10
DAS	5.11	C_19_H_26_O_7_	[M + NH_4_]^+^	384.20168	307.15329; 105.06977	15
FUS-X	4.28	C_17_H_22_O_8_	[M + Na]^+^	377.12073	228.16002; 175.07550	20
ZEN	6.55	C_18_H_22_O_5_	[M − H]^−^	317.13945	175.03989; 131.05008	−32
α-ZEL	4.87	C_18_H_24_O_5_	[M − H]^−^	319.15510	174.95630; 129.01947	36
β-ZEL	4.98	C_18_H_24_O_5_	[M − H]^−^	319.15510	174.95604; 160.97665	36
α-ZAL	4.81	C_18_H_26_O_5_	[M − H]^−^	321.17044	259.09497; 91.00272	29
β-ZAL	4.94	C_18_H_26_O_5_	[M − H]^−^	321.17044	259.09497; 91.00272	40
ZAN	5.00	C_18_H_24_O_5_	[M − H]^−^	319.15510	273.01187; 131.05020	35
BEA	5.77	C_45_H_57_N_3_O_9_	[M + NH_4_]^+^	801.44331	262.76715; 244.18239	70
ENN A	8.17	C_36_H_63_N_3_O_9_	[M + NH_4_]^+^	699.49026	228.15900; 210.14847	43
ENN A1	8.16	C_35_H_61_N_3_O_9_	[M + NH_4_]^+^	685.47461	228.15900; 210.14847	48
ENN B	7.87	C_33_H_57_N_3_O_9_	[M + NH_4_]^+^	657.44331	214.14320; 196.13280	50
ENN B1	8.06	C_34_H_59_N_3_O_9_	[M + NH_4_]^+^	671.45986	214.14343; 196.13295	48
AOH	5.88	C_14_H_10_O_5_	[M − H]^−^	257.04555	215.03490; 213.05569	−32
AME	6.82	C_15_H_12_O_5_	[M − H]^−^	271.06120	256.03751; 228.04276	−36

Abbreviations: Aflatoxins (AFB1, AFB2, AFG1 and AFG2), ochratoxin A (OTA), fumonisins (FB1 and FB2), deoxynivalenol (DON), 3-acetyl-deoxynivalenol (3-AcDON), 15-acetyl-deoxynivalenol (15-AcDON), HT-2 toxin, T-2 toxin, neosolaniol (NEO), diacetoxyscirpenol (DAS) fusarenon-X (FUS-X), zearalenone (ZEN), α-zearalenol (α-ZEL), β-zearalenol (β-ZEL), α-zearalanol (α-ZAL), β-zearalanol (β-ZAL), zearalanone (ZAN), beauvericin (BEA), enniatins (ENNA, ENNA1, ENNB and ENNB1), alternariol (AOH) and alternariol monomethyl ether (AME).

**Table 3:** Recent surveys reporting the occurrence of mycotoxins in pet foods samples.

Analyzed Samples (n)	Analytes Investigated	Mycotoxins	Positive Samples (%)	^a^ Range or Average (μg/kg)	Detection Methods	LOQ (μg/kg)	Reference
89	28	AFB1	25.8	3.3–7.9	UHPLC-Q-Orbitrap	0.013	This work
		AFB2	5.6	1.8–16.6		0.013	
		AFG1	1.1	11.1			
		AFG2	5.6	1.7–31.6		0.125	
		OTA	2.2	1.4–1.5		1.25	
		ZEN	91.0	0.9–60.6		0.013	
		Σ α + β-ZEL	87.6	0.9–58.9		α-ZEL = 1.25; β-ZEL = 0.125	
		Σ α + β -ZAL	79.8	<LOQ		α-ZAL = 1.25; β-ZAL = 0.125	
		ZAN	n.f.	n.f.		0.125	
		DON	30.3	7.6–297.3		1.25	
		Σ 3 + 15 AcDON	5.6	10.9–63.2		3-AcDON = 1.25; 15-AcDON = 1.25	
		NEO	n.f.	n.f.		0.188	
		HT2	32.6	3.3–110.1		1.25	
		T2	47.2	0.7–9.0		0.125	
		BEA	86.5	0.8–176.1		0.013	
		ENNA	10.1	0.3–9.6		0.125	
		ENNA1	22.5	0.4–28.1		0.125	
		ENNB	93.3	0.4–212.4		0.125	
		ENNB1	58.4	0.3–71.8		0.013	
		AOH	82.0	0.2–12.8		0.125	
		AME	84.3	0.1–15.6		0.125	
		FB1	66.3	11.8–990.1		0.125	
		FB2	52.8	10.5–556.3		0.250	
		DAS	n.f.	n.f.			
		FUS-X	n.f.	n.f.			
48	5	ENNA	n.a.	n.a.	LC-MS/MS	5	Tolosa et al., 2019 [16]
		ENNA1	41.5	8.1–11.9		1	
		ENNB	89	2.0–89.5		1	
		ENNB1	64	7.4–28.8		1	
		BEA	62	4.6–129.6		5	
32	8	AFB1	47.7	30.3–242.7	LC-MS/MS	1.7	Shao et al., 2018 [29]
		AFG1	13.9	13.9		0.7	
		OTA	16.2	15.1–17.3		10.7	
		ZEN	54.5	14.5–389.2		2.5	
		DON	66.3	22.8–421.3		16.5	
		T-2	15.4	15.4		3.3	
		BEA	19.1	0.2–153.4		2.5	
		FB1	87.2	6.6–191.9		10.0	
12	6	AFB1	n.a.	83.3	HPLC-FLD	41.57	Singh et al., 2017a [24]
		AFB2	n.a.	9.0		11.77	
		OTA	n.a.	1.0		-	
		ZEN	n.a.	5.7		-	
		FB1	n.a.	106.3		202.53	
		FB2	n.a.	61.9		118.37	
49	3	AFs	100	0.16–5.39	HPLC-FLD	AFB1 = 0.13; AFB2 = 0.59; AFG1 = 0.03; AFG2 = 0.22	Teixeira et al., 2017 [25]
		ZEN	95.9	4.07–98.3		3.95	
		FBs	77.6	37.4–1015		FB1 = 27.5; FB2 = 35.3	
100	4	AFLs	68	0.34–3.88	HPLC-FLD	B1=0.13; G1 = 0.03; B2 = 0.59; G2 = 0.22	Bissoqui et al., 2016 [23]
		ZEN	95	5.45–442.2		3.95	
		FB1	68	20.0–220		27.5	
		FB2	35	40.0–160		35.3	
20	3	AFs	n.a.	n.a.	ELISA-UV	5	Yasmina et al., 2016 [21]
		AFB1	15	2.6–18.4		1	
		OTA	70	2.62–6.65		2.5	
		ZEN	20	148–1170		1.75	
49	2	AFB1	8.2	<0.05–0.21	HPLC-FLD	0.15	Błajet-Kosicka et al., 2014 [22]
		OTA	46.9	<0.13–3		0.40	
	5	DON	100	22.7–436	LC-MS/MS	20.0	
		T-2	87.7	<0.5–13.3		1.50	
		HT-2	83.7	<1.60–19.6		5.00	
		ZEN	100	1.81–123		0.30	
7		FBs	28.6	<5–108		FB1 = 1.60; FB2 = 1.60; FB3 = 1.60	
76	4	DON	97	>250	ELISA-UV	-	Böhm et al., 2010 [30]
		OTA	5	3.5		-	
		ZEN	47	80		-	
		FBs	42	178		-	
29	3	DON	83	409	HPLC-FLD	25	
22		ZEN	68	185		20	
3		FBs	67	69		15	
180	1	AFB1	70.5	0.3–9.43	HPLC-FLD	0.1	Campos et al., 2008 [31]

Abbreviations: Aflatoxins (AFB1, AFB2, AFG1 and AFG2), ochratoxin A (OTA), fumonisins (FB1 and FB2), deoxynivalenol (DON), 3-acetyl-deoxynivalenol (3-AcDON), 15-acetyl-deoxynivalenol (15-AcDON), HT-2 toxin, T-2 toxin, neosolaniol (NEO), diacetoxyscirpenol (DAS) fusarenon-X (FUS-X), zearalenone (ZEN), α-zearalenol (α-ZEL), β-zearalenol (β-ZEL), α-zearalanol (α-ZAL), β-zearalanol (β-ZAL), zearalanone (ZAN), beauvericin (BEA), enniatins (ENNA, ENNA1, ENNB and ENNB1), alternariol (AOH) and alternariol monomethyl ether (AME). ^a^ Range or arithmetic mean of all positive samples. n.f. (not found) is shown if there was no readable value below LOD. n.a. not available.

**Table 4:** Nutritional composition of standard dry pet food samples.

Composition (%)	Dog (*n =* 55)	Cat (*n =* 34)
Proteins	25.8 ± 5.2	33.8 ± 3.8
Fats	14.9 ± 3.4	15.2 ± 4.4
Fibers	6.8 ± 1.2	7.5 ± 1.1
Total minerals	3.0 ± 2.1	3.9 ± 2.9

## Supplementary Materials

The following are available online at https://www.mdpi.com/2072-6651/11/8/434/s1, Table S1: Optimization of sample preparation procedure, Table S2: Accuracy and precision of the developed method.
