# Supplementary material for: Target Analysis and Retrospective Screening of Multiple Mycotoxins in Pet Food Using UHPLC-Q-Orbitrap HRMS
Source: Toxins (Basel). 2019 Jul 24;11(8):434. doi: 10.3390/toxins11080434 (PMC6723864; doi:10.3390/toxins11080434)
Supplement: Supplementary file 1 [file toxins-11-00434-s001.pdf]

Supplementary Materials: Target Analysis and Retrospective Screening of Multiple Mycotoxins in Pet Food Using UHPLC-Q-Orbitrap HRMS

Luigi Castaldo, Giulia Graziani, Anna Gaspari, Luana Izzo, Josefa Tolosa, Yelko Rodríguez-Carrasco and Alberto Ritieni

Table S1. Optimization of sample preparation procedure. Spiking level: 20 μg/kg.

Analyte	Recovery ± RSD (%; *n* = 3)						
	Stirring time			Sonication treatment		Clean up	
	15 min	30 min	60 min	without	15 min	Freeze-out (2h)	$MgSO_4$ + C18
AFB1	27 ± 16	49 ± 14	75 ± 8	64 ± 15	75 ± 11	65 ± 14	81 ± 8
AFB2	30 ± 19	51 ± 12	72 ± 9	68 ± 10	79 ± 8	69 ± 13	86 ± 11
AFG1	24 ± 14	53 ± 11	79 ± 8	61 ± 14	73 ± 12	67 ± 14	88 ± 9
AFG2	32 ± 17	48 ± 16	74 ± 9	58 ± 13	72 ± 9	62 ± 15	91 ± 12
OTA	36 ± 21	55 ± 15	80 ± 10	71 ± 16	81 ± 14	79 ± 10	98 ± 9
FB1	21 ± 12	66 ± 9	79 ± 12	59 ± 21	86 ± 11	74 ± 12	76 ± 12
FB2	18 ± 15	69 ± 10	80 ± 11	62 ± 19	91 ± 12	70 ± 11	79 ± 10
DON	25 ± 19	70 ± 16	86 ± 14	81 ± 18	94 ± 10	81 ± 16	81 ± 11
3-ADON	19 ± 11	73 ± 16	80 ± 12	79 ± 20	96 ± 14	76 ± 14	77 ± 12
15-ADON	21 ± 13	67 ± 8	91 ± 11	83 ± 17	90 ± 8	83 ± 12	79 ± 9
HT-2	35 ± 21	72 ± 17	95 ± 9	77 ± 19	84 ± 10	94 ± 9	93 ± 9
T-2	39 ± 18	74 ± 19	97 ± 7	81 ± 21	80 ± 11	90 ± 7	99 ± 7
NEO	14 ± 10	65 ± 9	98 ± 10	84 ± 18	93 ± 13	97 ± 10	95 ± 10
DAS	24 ± 11	73 ± 13	91± 12	82 ± 15	94 ± 9	76 ± 12	97 ± 10
FUS-X	19 ± 13	71 ± 13	87 ± 10	79 ± 18	92 ± 10	74 ± 12	86 ± 12
ZEN	20 ± 10	65 ± 12	94 ± 12	81 ± 14	90 ± 11	62 ± 15	94 ± 8
α-ZEL	26 ± 13	69 ± 15	92 ± 15	75 ± 19	89 ± 14	68 ± 15	82 ± 11
β-ZEL	24 ± 17	71 ± 15	86 ± 14	79 ± 16	84 ± 8	64 ± 116	75 ± 12
α-ZAL	17 ± 12	70 ± 9	94± 12	83 ± 16	83 ± 8	71 ± 14	79 ± 10
β-ZAL	19 ± 15	70 ± 8	90 ± 13	84 ± 18	94 ± 13	69 ± 12	76 ± 10
ZAN	23 ± 14	72 ± 12	88 ± 11	89 ± 14	82 ± 8	73 ± 13	77 ± 11
BEA	35 ± 23	78 ± 21	105 ± 12	77 ± 13	96 ± 7	94 ± 9	98 ± 6
ENN A	36 ± 12	75 ± 19	90 ± 16	89 ± 12	94 ± 9	96 ± 7	92 ± 8
ENN A1	40 ± 15	74 ± 16	97 ± 9	84 ± 10	97 ± 7	91 ± 10	93 ± 8
ENN B	38 ± 12	78 ± 10	95 ± 9	80 ± 9	105 ± 10	97 ± 9	93 ± 9
ENN B1	34 ± 10	70 ± 9	93 ± 7	79 ± 14	109 ± 9	93 ± 8	97 ± 7
AOH	27 ± 9	67 ± 10	84 ± 8	81 ± 17	112 ± 11	83 ± 14	108 ± 12
AME	32 ± 11	72 ± 8	89 ± 9	83 ± 20	114 ± 13	87 ± 15	112 ± 10

Aflatoxins (AFB1, AFB2, AFG1 and AFG2), ochratoxin A (OTA), fumonisins (FB1 and FB2), deoxynivalenol (DON), 3-acetyl-deoxynivalenol (3-AcDON), 15-acetyl-deoxynivalenol (15-AcDON), HT-2 toxin, T-2 toxin, neosolaniol (NEO), diacetoxyscirpenol (DAS) fusarenon-X (FUS-X), zearalenone (ZEN), α-zearalenol (α-ZEL), β-zearalenol (β-ZEL), α-zearalanol (α-ZAL), β-zearalanol (β-ZAL), zearalanone (ZAN), beauvericin (BEA), enniatins (ENNA, ENNA1, ENNB and ENNB1), alternariol (AOH) and alternariol monomethyl ether (AME).

Table S2. Accuracy and precision of the developed method.

Analyte	Accuracy, (Recovery (%))			Precision, (RSD_r, % (RSD_R, %))		
	10 μg/kg	20 μg/kg	50 μg/kg	10 μg/kg	20 μg/kg	50 μg/kg
AFB1	76	81	87	9 (11)	8 (11)	9 (13)
AFB2	79	86	84	10 (13)	11 (13)	4 (7)
AFG1	80	88	96	7 (10)	9 (10)	7 (9)
AFG2	84	91	97	8 (8)	12 (16)	6 (8)
OTA	86	98	106	11 (12)	9 (13)	8 (11)
FB1	75	76	86	9 (14)	12 (15)	4 (9)
FB2	75	79	90	8 (10)	10 (11)	9 (10)
DON	80	81	96	7 (9)	11 (14)	8 (11)
3-ADON	75	77	94	9 (10)	12 (15)	7 (8)
15-ADON	81	79	89	10 (12)	9 (10)	6 (10)
HT-2	86	93	94	11 (10)	9 (14)	5 (9)
T-2	94	99	102	8 (14)	7 (12)	8 (11)
NEO	90	95	99	7 (12)	10 (14)	9 (14)
DAS	86	97	106	6 (9)	10 (17)	8 (10)
FUS-X	81	86	96	9 (15)	12 (15)	9 (11)
ZEN	79	94	97	8 (12)	8 (16)	10 (15)
α-ZEL	78	82	95	10 (13)	11 (14)	11 (15)
β-ZEL	77	75	91	12 (16)	12 (15)	9 (16)
α-ZAL	75	79	86	7 (11)	10 (14)	10 (13)
β-ZAL	79	76	87	9 (13)	10 (12)	8 (12)
ZAN	75	77	85	8 (10)	11 (13)	7 (10)
BEA	90	98	101	7 (12)	6 (9)	6 (14)
ENN A	94	92	99	11 (15)	8 (12)	9 (14)
ENN A1	92	93	98	12 (12)	8 (10)	8 (15)
ENN B	88	93	103	8 (11)	9 (13)	10 (13)
ENN B1	91	97	105	9 (11)	7 (15)	11 (15)
AOH	96	108	105	10 (13)	12 (16)	8 (13)
AME	99	112	109	8 (10)	10 (17)	7 (12)

RSD_r: intra-day precision (repeatability; n = 3); RSD_R: inter-day precision (within-laboratory repeatability; n = 9): Aflatoxins (AFB1, AFB2, AFG1 and AFG2), ochratoxin A (OTA), fumonisins (FB1 and FB2), deoxynivalenol (DON), 3-acetyl-deoxynivalenol (3-AcDON), 15-acetyl-deoxynivalenol (15-AcDON), HT-2 toxin, T-2 toxin, neosolaniol (NEO), diacetoxyscirpenol (DAS) fusarenon-X (FUS-X), zearalenone (ZEN), α-zearalenol (α-ZEL), β-zearalenol (β-ZEL), α-zearalanol (α-ZAL), β-zearalanol (β-ZAL), zearalanone (ZAN), beauvericin (BEA), enniatins (ENNA, ENNA1, ENNB and ENNB1), alternariol (AOH) and alternariol monomethyl ether (AME).
